# Addendum to ‘Revision of the Late Jurassic teleosaurid genus *Machimosaurus* (Crocodylomorpha, Thalattosuchia)’

**DOI:** 10.1098/rsos.150024

**Published:** 2015-02-25

**Authors:** Mark T. Young, Stéphane Hua, Lorna Steel, Davide Foffa, Stephen L. Brusatte, Silvan Thüring, Octávio Mateus, José Ignacio Ruiz-Omeñaca, Philipe Havlik, Yves Lepage, Marco Brandalise de Andrade

**Affiliations:** 1 School of GeoSciences, University of Edinburgh, The King's Buildings, Edinburgh EH9 3JFE, UK; 2 School of Ocean and Earth Science, National Oceanography Centre, University of Southampton, Southampton SO14 3ZH, UK; 3 Le Musée des Dinosaures d'Espéraza, Espéraza 11260, France; 4 Department of Earth Sciences, Natural History Museum, London SW7 5BD, UK; 5 School of Earth Sciences, University of Bristol, Wills Memorial Building, Bristol BS8 1RJ, UK; 6 National Museums Scotland, Chambers St., Edinburgh EH1 1JF, UK; 7 Naturmuseum Solothurn, Klosterplatz 2, Solothurn 4500, Switzerland; 8 GeoBioTec, Departamento de Ciências da Terra, Faculdade de Ciências e Tecnologia, Universidade Nova de Lisboa, Quinta da Torre, Caparica 2829-516, Portugal; 9 Museu da Lourinhã, Rua João Luis de Moura 9, Lourinhã 2530-158, Portugal; 10 Museo del Jurásico de Asturias (MUJA), Colunga 33328, Spain; 11 Paläontologische Sammlung, Senckenberg Center for Human Evolution and Palaeoenvironment, Eberhard Karls Universität, Sigwartstrasse 10, Tübingen 72072, Germany; 12 Sciences et Géologie Normandes, Le Havre 76620, France; 13 Faculdade de Biociências, Pontifícia Universidade Católica do Rio Grande do Sul, Av. Ipiranga 6681, 90619-900 Porto Alegre, Brazil

## 1. Introduction

This addendum is to ensure that the ICZN (International Commission on Zoological Nomenclature) Code criteria for the availability of new nomenclatural acts are satisfied. In our original publication [1], the newly established taxon *Machimosaurus* *buffetauti* was not named in accordance with the ICZN Code, specifically it failed to fulfil Article 8.5.3. This addendum, being a ‘corrigendum’ in the sense of the Glossary of the Code, should be consulted alongside the original publication [1]. However, it is this addendum that fulfils the ICZN Code criteria for establishing the name *M. buffetauti*. As such, the date of publication and nominal authority of that nomenclatural act is the date that this addendum has been published.

This addendum fulfils Article 8.5.3 of the ICZN Code, namely to ‘be registered in the *Official Register of Zoological Nomenclature* (ZooBank) (see Article 78.2.4)’. Thus, we have officially registered the new nomenculatural act *M. buffetauti* (in addition to registering the genus, type species and the publication establishing them). Note that at present (14/Jan/2015), ZooBank only accepts new names, as other nomenclatural acts (such as amendment of spellings) are not currently supported. As such, *Machimosaurus* is currently registered under *Madrimosaurus* (see [1] for details). Below are the resultant LSIDs (Life Science Identifiers), and we provide an updated Systematic Palaeontology section from Young *et al*. [1] thus ensuring *M. buffetauti* sp. nov. is now a valid name.

In addition to being registered with ZooBank, and having the LSIDs recorded within, the electronic edition of this work was published in a journal with an ISSN, and has been archived and is available from the following digital repositories: CLOCKSS and PORTICO.

**Institutional abbreviations.** DFMMh, Dinosaurier-Freilichtmuseum Münchehagen, Lower Saxony, Germany; GPIT, Paläontologische Sammlung der Eberhard Karls Universität Tübingen, Germany; MPV, Musée paléontologique (Paléospace) de Villers-sur-Mer, Normandy, France; NHMUK, Natural History Museum, London, United Kingdom; SMNS, Staatliches Museum für Naturkunde Stuttgart, Germany.

## 2. Zoobank LSIDs

**Original publication (Young *et al*. [1])**
urn:lsid:zoobank.org:pub:9125E890-E1E3–4FF8-BB24-D693DFE4F286
**This addendum**
urn:lsid:zoobank.org:pub:E4733A09-2781–43BE-8F2C-621D5C29B7C6
**The publication von Meyer, 1837 [2]**
urn:lsid:zoobank.org:pub:7B719821-F101–4380-A590-12E2D86704EC
**Genus** ***Machimosaurus*** **(note that it is registered under the original mis-spelling of** ***Madrimosaurus*** **von Meyer, 1837 [2])**
urn:lsid:zoobank.org:act:DEB4DD43–173A-4B21-B4F0–1C0075B30C3D
**Type species,** ***Machimosaurus hugii*** [2]
urn:lsid:zoobank.org:act:D44630DF-5C84–4A73–8D13-A2CF4F4948C5
**New Species**, ***Machimosaurus buffetauti***
urn:lsid:zoobank.org:act:EAA53B27–2A4C-4395-B776-CDCFD715C413

## 3. Systematic palaeontology

***Machimosaurus*** **von Meyer, 1837 [2]** **emend. von Meyer, 1838** [3]

*Machimosaurus buffetauti* sp. nov.

— v 1873 *Steneosaurus burgensis*nomen nudum—Jarrin [4]
— v 1876 *Steneosaurus burgensis*nomen nudum—Jarrin [5]
— v 1905 *Steneosaurus burgensis chanuti* nomen nudum—Chanel [6, figs. 1–3]
— v 1982 *Machimosaurus hugii*von Meyer—Buffetaut [7, Plate 1, figs. A–D]
— v 1982 *Machimosaurus hugii*von Meyer—Buffetaut [8, Plate 1]
— v 2004 *Machimosaurus hugii*von Meyer—Karl & Tichy [9, figs. 1–2]
— v 2006 *Machimosaurus hugii*von Meyer—Karl *et al*. [10, fig. 8]
— v 2008 *Machimosaurus hugii*von Meyer—Lepage *et al*. [11, figs. 1–7]
— v 2008 *Machimosaurus hugii*von Meyer—Pierce *et al*. (*partim*) [12]
— v 2013 *Machimosaurus hugii*von Meyer—Martin & Vincent [13, figs. 1–9]
— v 2014 *Machimosaurus buffetauti* sp. nov. et invalid—Young *et al*. [1, figs. 1–9]
— v 2014 *Machimosaurus buffetauti*invalid name—Young *et al*. [14, figs. 2–6]

**Holotype.** SMNS 91415: complete skull and mandible, with partial post-cranial skeleton ([1, figs. 22–27], [13, figs. 1–9] and [14, fig. 2]). The holotype repository is the Staaliches Museum für Naturkunde Stuttgart.

**Holotype locality.** Am Hörnle Quarry, Neuffen, Baden-Württemberg, Germany [13].

**Holotype horizon.** Lacunosamergel Formation, *Ataxioceras hypselocyclum* Sub-Mediterranean ammonite Zone (Weißer Jura gamma 2), lower Kimmeridgian, Upper Jurassic [13].

**Etymology.** ’Buffetaut's pugnacious lizard’. Named in honour of Eric Buffetaut (b. 1950), whose research has greatly elucidated thalattosuchian and crocodylomorph evolution.

**Referred specimens.** MPV V.1600.Bo and V.1601.Bo—anterior half of rostrum (premaxilla, maxilla and dentary) in occlusion and a maxilla-nasal fragment (Calcaires Coquilliers Formation; *Pictonia baylei* Sub-Boreal ammonite Zone, lowermost Kimmeridgian of Cricqueboeuf, Normandy, Northern France [1, figs. 12–14]) [7,11].

MPV KIM027—Cricqueboeuf, Normandy, France (Calcaires à *Harpagodes* Member of the Marnes de Bléville Formation, *Rasenia cymodoce* Sub-Boreal ammonite Zone, lower Kimmeridgian [14, fig. 5]).

DFMMh FV 330, DFMMh FV 541: isolated tooth crowns (Langenberg Formation; Langenberg near Oker, Lower Saxony, Germany; Kimmeridgian [1, fig. 21C-H] and [14, figs. 3–4]) [9,10].

Musée de Brou (Bourg-en-Bresse, France), specimen number unknown—a complete skull and mandible in articulation (Calcaires à ptérocères Formation, lower Kimmeridgian; Montmerle, Bourg-en-Bresse, département de l'Ain, France [8]).

**Geological range.** Lower Kimmeridgian.

**Geographical range.** Europe (France and Germany). An isolated tooth from Smallmouth Sands, England (NHMUK PV R1774 [1, fig. 21A-B] and [14, fig. 6]) is possibly referable to this taxon, as are isolated teeth from Czarnogłowy, Poland (GPIT/RE/328, GPIT/RE/9280 and GPIT/RE/9281 [1, figs. 31–32]).

**Species diagnosis.** Teleosaurid crocodylomorph within the genus *Machimosaurus* with the following unique combination of characters (autapomorphic characters are indicated by an asterisk *): 21–22 alveoli per maxilla (approx. 16–17 of which are anterior to the palatines); 24/25 alveoli per dentary (19–20 of which are adjacent to the mandibular symphysis); low post-symphyseal dentary tooth count (2 pairs)*; inter-alveolar spaces between the maxillary and dentary alveoli are variable in size (thalattosuchian symplesiomorphy); orbits are sub-circular in shape (transverse and anteroposterior axes are sub-equal; the *Steneosaurus brevior* holotype also has circular orbits)*; the quadrates have a single large circular depression on the dorsal surface near the hemicondyles*; basioccipital apophysis has a ‘U-shaped’ cross-section (teleosaurid symplesiomorphy); the inter-basioccipital tubera notch is a wide and gentle inverse semi-circle when seen in occipital view (teleosaurid symplesiomorphy); basioccipital tuberosities (basal tubera) are reduced in size when seen in occipital view (apomorphy shared with *Machimosaurus mosae*); axis neural arch dorsal margin is strongly concave when seen in lateral view*; axis postzygapophyses terminate significantly posterior to posterior-surface of the centrum view (somewhat similar to that seen in *Steneosaurus durobrivensis*); coracoid glenoid process (process near the glenoid fossa that projects posterodorsally) is elongate, extending considerably from the head of the coracoid, and is a sub-isosceles triangle in shape when seen in lateral view*; coracoid postglenoid process anterior margin is very slightly concave, and terminates approximately in the same frontal plane as the glenoid*; coracoid postglenoid process posterior margin is strongly concave, and terminates approximately in the same frontal plane as the posterior-end of the glenoid process*; dorsal osteoderm ornamentation is composed of small-to-large irregularly shaped pits arranged in a random manner, that are well separated from one another (somewhat similar to *Steneosaurus leedsi*).

***Steneosaurus burgensis***. The name *S. burgensis*, and *S. burgensis chanuti*, have been applied to the *Machimosaurus* skull from Ain, France [4–6,8]. These specific and sub-specific names are however *nomina nuda*. Both Jarrin [5] and Chanel [6] stated that the Ain skull was sent to Caen for preparation and study by Eugène Eudes-Deslongchamps, who proposed the name *Steneosaurus burgensis* for the specimen, in consultation with the Société d'émulation de l'Ain. Neither Jarrin [4,5] nor Chanel [6] established the name under Article 12 of the ICZN Code as: they did not describe the specimen, nor did they provide a definition of the species; they simply reported that the name was proposed by Eudes-Deslongchamps. Unfortunately, Eudes-Deslongchamps never published his description of the Ain skull [6,8]. The specimen was not formally described until 1982, and then it was referred to *Machimosaurus hugii* [8]. The sub-specific epithet *chanuti* was apparently established by those who did not fully understand zoological nomenclature [8], as it was added to the Ain skull's specimen plaque solely to honour the discoverer [6,8].

Our decision to establish a new taxon based on SMNS 91415, and not formally establish *S. burgensis* for the Ain skull was for several reasons: (i) the Ain skull is partially reconstructed, and it is unclear how much is plaster and how much is real bone [8]; (ii) the cranium and lower jaw of the Ain skull are in articulation, meaning that the palatal and dorsal mandibular morphologies cannot be seen [6,8]; (iii) the German skull SMNS 91415 has the cranium and lower jaw disarticulated, allowing these morphologies to be observed [13] and (iv) SMNS 91415 has associated post-cranial material, greatly aiding in comparisons with other *Machimosaurus* taxa, in particular, *M. mosae*.
